# A dataset of ^137^Cs activity concentration and inventory in forests contaminated by the Fukushima accident

**DOI:** 10.1038/s41597-020-00770-1

**Published:** 2020-12-18

**Authors:** Shoji Hashimoto, Naohiro Imamura, Ayumi Kawanishi, Masabumi Komatsu, Shinta Ohashi, Kazuya Nishina, Shinji Kaneko, George Shaw, Yves Thiry

**Affiliations:** 1 Department of Forest Soils, Forestry and Forest Products Research Institute, Tsukuba, Ibaraki 305-8687 Japan; 2 Graduate School of Agricultural and Life Sciences, The University of Tokyo, Bunkyo-ku, Tokyo 113-8657 Japan; 3 Department of Mushroom Science and Forest Microbiology, Forestry and Forest Products Research Institute, Tsukuba, Ibaraki 305-8687 Japan; 4 Department of Wood Properties and Processing, Forestry and Forest Products Research Institute, Tsukuba, Ibaraki 305-8687 Japan; 5 Center for Regional Environmental Research, National Institute for Environmental Studies, Tsukuba, Ibaraki, 305-8506 Japan; 6 Kansai Research Center, Forestry and Forest Products Research Institute, Fushimi, Kyoto 612-0855 Japan; 7 School of Biosciences, University of Nottingham, Sutton Bonington, Nottingham, LE12 5RD United Kingdom; 8 Research and Development Division, Andra, 1-7 Rue Jean-Monnet, 92298 Châtenay-Malabry cedex, France

**Keywords:** Environmental impact, Element cycles

## Abstract

The majority of the area contaminated by the Fukushima Daiichi Nuclear Power Plant accident is covered with forests. We developed a dataset for radiocaesium (^137^Cs) in trees, soil, and mushrooms measured at numerous forest sites. The ^137^Cs activity concentration and inventory data reported in scientific journal papers written in English and Japanese, governmental reports, and governmental monitoring data on the web were collated. The ancillary information describing the forest stands were also collated, and further environmental information (e.g. climate) was derived from the other databases using longitude and latitude coordinates of the sampling locations. The database contains 8593, 4105, and 3189 entries of activity concentration data for trees, soil, and mushrooms, and 471 and 3521 entries of inventory data for trees and soil, respectively, which were collected from 2011 to 2017, and covers the entire Fukushima prefecture. The data can be used to document and understand the spatio-temporal dynamics of radiocaesium in the affected region and to aid the development and validation of models of radiocaesium dynamics in contaminated forests.

## Abstract (summary)

**Table:** 

Measurement(s)	activity (of a radionuclide) • Cesium Cs 137
Technology Type(s)	digital curation
Factor Type(s)	geographic location
Sample Characteristic - Organism	Cryptomeria japonica • Chamaecyparis obtusa • Pinus densiflora • Quercus serrata
Sample Characteristic - Environment	temperate evergreen needleleaf forest • temperate deciduous broadleaf forest • leaf • wood • litter layer • forest soil • mushroom (food source) • bark • pollen
Sample Characteristic - Location	Japan • Fukushima Prefecture

Machine-accessible metadata file describing the reported data: 10.6084/m9.figshare.13166462

## Background & Summary

The Fukushima Daiichi Nuclear Power Plant (FDNPP) accident in 2011 is the largest nuclear disaster since the Chernobyl Nuclear Power Plant accident in 1986. Radioactive materials leaked, and spread widely across Japan, especially in the north west region of the FDNPP. The weather conditions led to heterogeneous atmospheric deposits of radioactive materials, in particular caesium-134 (^134^Cs) and caesium-137 (^137^Cs), in eastern Japan. The largest landcover in the area contaminated by the FDNPP radioactive fallout is forest^1^. Since just after the accident, much research and monitoring of radiocaesium in forests has been conducted, and these studies have reported many data. However, these data were published in various media, for example, scientific papers both in international journals written in English and in domestic journals written in Japanese. Also, the Japanese government and the local governments extensively collected data of radioactivity in contaminated forests, and those data were published in their domestic reports, which are not easily traceable. Furthermore, there was a specific dataset for monitored radioactivity in mushrooms that was made available on the web by the Japanese government.

Research in Chernobyl and Fukushima revealed that radiocaesium deposited on forests continues to migrate within forests^2–5^. At the time of deposition the leaves and branches of trees intercepted radiocaesium directly, then the captured radiocaesium moved first to the forest floor via rainfall and litterfall, and then further down into the mineral soil as time progressed. A part of the radiocaesium is absorbed in tree tissues through the tree surfaces (leaves and bark) and root uptake. These transfers differ between locations and combinations of forest/tree/soil types (e.g. evergreen or deciduous)^6,7^.

In addition, trees consist of various physiologically different parts, such as leave, branches, bark, wood, and roots; furthermore, forest systems include not only trees, but also soil surface organic layers, mineral soils, mushrooms, and various animals. The wood of trees is the most important part of the ecosystem in terms of forestry products, but to trace the dynamics of radiocaesium within forests, tracking the radiocaesium in major functional compartments within forests is essential.

Therefore, to document the radioactive contamination of forests, and to capture the more representative situations of forest contamination, a comprehensive dataset is essential^8–12^. To that end, in this database we aimed to provide a database that can be used to document changes in the spatio-temporal distributions of radiocaesium in the affected region, to better understand the fate of radiocaesium dynamics in forests, and to aid the development and validation of models of radiocaesium dynamics in forests.

We collated the ^137^Cs activity concentration and inventory data (^137^Cs activity per unit ground area) reported in journal papers written in English or in Japanese, governmental reports, and monitoring data on the web provided by the government. We further collated the ancillary site information from the source and those derived from the other databases linked with location.

The database contains 8593, 4105, and 3189 entries of activity concentration data for trees, soil, and mushrooms, and 471 and 3521 entries of inventory data for trees and soil, respectively, which were observed from 2011 to 2017, and in particular intensively covers the entire Fukushima prefecture. The data for mushrooms were taken across the wide range of eastern Japan including the Fukushima prefecture. As for tree species, Sugi cedar (*Cryptomeria japonica*), which is the most important plantation tree species in Japan, was most intensively investigated, and data for Hinoki cypress (*Chamaecyparis obtusa*), pine (*Pinus densiflora*), and oak (mainly *Quercus serrata*) were abundant too.

This database is a precious resource to refine our knowledge on biogeochemical cycling of radiocaesium within forests, and also represents a good basis to bridge disciplines and to develop interdisciplinary environmental studies.

## Methods

We collected all related research studies in the peer-reviewed scientific literature using the Web of Science (https://www.webofknowledge.com/), and J-Stage (https://www.jstage.jst.go.jp/). The search terms we used were “Fukushima”, “forests”, and “cesium/caesium/radiocesium/radiocaesium”. The studies written in English were searched using the Web of Science, and those in Japanese were searched using the J-Stage. We further collected reports of Japan’s governmental and local governmental projects conducted in the Fukushima prefecture. We collated radiocaesium activity concentration and inventory data together with the ancillary data of forest stand and location of the study sites. As for the mushroom data, we collated data from the governmental web pages for publishing monitoring data. The original values were decay-corrected to the sampling date where provided. When the values were lower than detection limits, the values were shown with the inequality sign. When only the total radioactivity of ^134^Cs and ^137^Cs were reported, we estimated the radioactivity of ^137^Cs by decay correction with the assumption that the ratio of ^134^Cs: ^137^Cs on 11 March 2011 was 1:1. Data for animals are not the major target of the database, but when we found a reference, we incorporated these data in the database. When data were shown only in plots, we extracted values by measuring points/bars in the plots using software (GSYS2.4, JCPRG, Japan). When longitude and latitude coordinates were provided, we combined ancillary data using the other databases: distance from the power plant, air-borne survey based air dose rate, ^137^Cs, and ^134^Cs deposition^13^, annual mean air temperature, precipitation, elevation, and soil type^14^. For mushroom records, ecosystem types (e.g. litter/wood decomposing) were added.

The database consists of three separate files: 1) a data file, which contains radioactivity and ancillary data, 2) a field-description file, which explains the contents of the data file and the units of data, and 3) a reference list file, which contains the source number and the details of the reference. When the source has only a Japanese title, we translated the title into English, and included both titles in the reference list file.

## Data Records

**Fig. 1:**
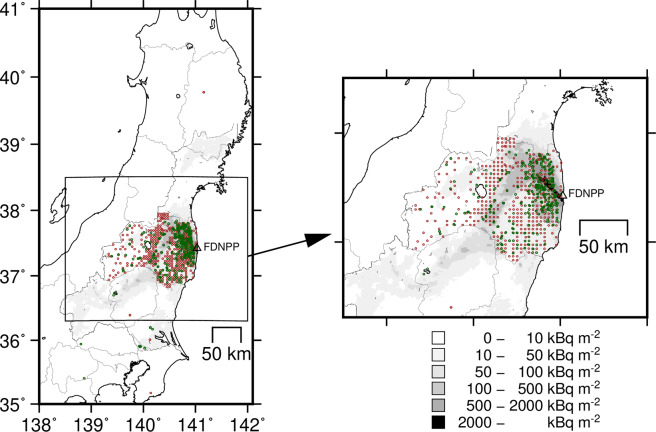
The map of sampling sites. Green circles show the sampling sites for trees, and brown open circles show the sampling sites for soil. For mushrooms, no longitude and latitude coordinates were provided in the source. A white triangle is the location of Fukushima Daiichi Nuclear Power Plant (FDNPP), and the grey base map is the air-borne based ^137^Cs map (the fifth survey)^13^. Please note that longitude and latitude coordinates for a part of data entries are not reported in the source even for trees and soil, and hence not all sampling sites are shown on the map.

The dataset, in spreadsheet format (Microsoft Excel), can be found in the ZENODO repository with the title “^137^Cs in forest ecosystems contaminated by the Fukushima Daiichi Nuclear Power Plant Accident”^15^. The creators are: Shoji Hashimoto, Naohiro Imamura, Ayumi Kawanishi, Masabumi Komatsu, Shinta Ohashi, Kazuya Nishina, Shinji Kaneko, George Shaw, Yves Thiry. A summary table of the number of records by sampling year and items are presented in Table 1. In total, we extracted 16480 entries: 16136 activity concentration data and 3992 inventory data (3668 data entries have both activity concentration and inventory). The sampling years of the records ranged from 2011 to 2017, and the sampling sites cover the entire Fukushima prefecture (Fig. 1). The data for mushrooms were taken across the wide range of eastern Japan, 14 prefectures including Fukushima prefecture. For mushrooms, the data have no longitude and latitude coordinates but only the municipality’s name for sampling locations. The database contains 8593, 4105, and 3189 entries of activity data for trees, soil, and mushrooms, and 471 and 3521 entries of inventory data for trees and soil, respectively. As for tree species, Sugi cedar (*Cryptomeria japonica*), which is the most important plantation tree species in Japan, was most intensively investigated, and data for Hinoki cypress (*Chamaecyparis obtusa*), pine (*Pinus densiflora*), and oak (mainly *Quercus serrata*) were rich (Table 2).

**Table 1:** Summary of counts for activity concentration and inventory data entry.

Sampling year	Activity concentration				Inventory	
	Tree	Soil	Mushroom	Animal	Tree	Soil
2011	578	1120	151	55	80	1001
2012	2070	851	333	149	76	527
2013	1148	417	508	45	120	381
2014	2172	622	592	0	84	616
2015	1376	591	632	0	99	569
2016	1071	420	566	0	4	369
2017	178	84	407	0	8	58
Total	8593	4105	3189	249	471	3521

**Table 2:** Summary of counts for activity concentration data entry for major tree parts among tree species.

Part	Species					Total
	Cedar (*Cryptomeria japonica*)	Cypress (*Chamaecyparis obtusa*)	Pine (*Pinus densiflora*)	Oak (mainly *Quercus serrata*)	Other	
Leaf	1081	194	240	118	350	1983
Branch	251	109	149	172	105	786
Bark	360	117	185	151	31	844
Inner bark	82	36	22	28	20	188
Outer bark	416	120	252	38	21	847
Wood	267	85	126	45	5	528
Heartwood	609	182	328	193	34	1346
Sapwood	621	190	344	215	48	1418
Pollen	115	0	0	0	0	115
Other	290	2	3	216	27	538
Total	4092	1035	1649	1176	641	8593

## Technical Validation

The data validation was conducted in two ways. Firstly, all data entries were double checked by a researcher who had not made the primary data entry. Secondly, for data with longitude and latitude coordinates, mistakes in data entry were detected by plotting data with total deposition ratio: the total deposition information was derived using the location information. In general, activity concentrations are positively correlated with total deposition; hence, we identified the outliers visually, and checked the data entries for outliers with the original source and modified the data entry errors. Obvious errors of longitude and latitude coordinates were validated by plotting the sampling location on a map. The mushroom data on the web contained a certain number of typographic errors and inconsistencies in names, which we cleaned up manually.

## Usage Notes

Some data reported in different journal papers were often overlapping (ie. reported twice). The master data records and the duplications were identified in the “Flag_duplication”. To simply remove the duplication and extract the master data only, use the data with no flag. The sources of duplicated data are shown in “Related_references”, so the master data can be changed depending on the purpose of the study. The litter and soil data from the same soil vertical profile were identified in the “Soil_profile_number”. For example, to sum the total inventory of a soil profile or draw a vertical distribution in soil, you can use this identifier.
