# Author Correction: A dataset of ^137^Cs activity concentration and inventory in forests contaminated by the Fukushima accident

**DOI:** 10.1038/s41597-021-00855-5

**Published:** 2021-03-03

**Authors:** Shoji Hashimoto, Naohiro Imamura, Ayumi Kawanishi, Masabumi Komatsu, Shinta Ohashi, Kazuya Nishina, Shinji Kaneko, George Shaw, Yves thiry

**Affiliations:** 1 Department of Forest Soils, Forestry and Forest Products Research Institute, Tsukuba, Ibaraki 305-8687 Japan; 2 Graduate School of Agricultural and Life Sciences, The University of Tokyo, Bunkyo-ku, Tokyo, 113-8657 Japan; 3 Department of Mushroom Science and forest Microbiology, Forestry and Forest Products Research Institute, Tsukuba, Ibaraki 305-8687 Japan; 4 Department of Wood Properties and Processing, Forestry and Forest Products Research Institute, Tsukuba, Ibaraki 305-8687 Japan; 5 Center for Regional Environmental Research, National Institute for Environmental Studies, Tsukuba, Ibaraki 305-8506 Japan; 6 Kansai Research Center, Forestry and Forest Products Research Institute, Fushimi, Kyoto, 612-0855 Japan; 7 School of Biosciences, University of Nottingham, Sutton Bonington, Nottingham, LE12 5RD UK; 8 Research and Development Division, Andra, 1-7 Rue Jean-Monnet, 92298 Châtenay-Malabry cedex, France

**Keywords:** Environmental impact, Element cycles

Correction to: *Scientific Data* 10.1038/s41597-020-00770-1, published online 18 December 2020

Figure 1 has been updated to include the correct unit of the background map, kBq m^-2^. This error has been corrected in both the HTML and PDF versions of this Data Descriptor.

